# Expression of Concern: STEAP1 facilitates metastasis and epithelial-mesenchymal transition of lung adenocarcinoma via the JAK2/STAT3 signaling pathway

**DOI:** 10.1042/BSR-2019-3169_EOC

**Published:** 2023-05-23

**Keywords:** EMT, JAK2/STAT3 signaling, Lung adenocarcinoma, STEAP1

The Editorial Office has been made aware of potential issues surrounding the scientific validity of this paper, hence has issued an expression of concern to notify readers whilst the Editorial Office investigates. The authors contacted the Editorial Office regarding a duplication in Figure 3C between the A549 migration vector image and the invasion vector image. A corrected image, which also includes the replacement of the Figure 3C STEAP1 panels, has been provided and is under review by the Editorial Board, and communication remains in progress regarding the western blot data of the figures and the raw data provided by the authors.

